# Stress Mindset and Social Identification in Chronic Pain Patients and Their Relationship to Coping, Well-Being & Depression

**DOI:** 10.1007/s10880-022-09883-8

**Published:** 2022-05-16

**Authors:** Isabel Grünenwald, Antonia J. Kaluza, Martin Schultze, Rolf van Dick

**Affiliations:** Department of Psychology, Goethe University Frankfurt, Theodor-W.-Adorno-Platz 6, 60323 Frankfurt, Germany

**Keywords:** Stress mindset, Social identity, Chronic pain, Pain patients, Coping

## Abstract

We predicted that chronic pain patients have a more negative stress mindset and a lower level of social identification than people without chronic pain and that this, in turn, influences well-being through less adaptive coping. 1240 participants (465 chronic pain patients; 775 people in the control group) completed a cross-sectional online-survey. Chronic pain patients had a more negative stress mindset and a lower level of social identification than people without chronic pain. However, a positive stress mindset was linked to better well-being and fewer depressive symptoms, through the use of the adaptive coping behaviors *positive reframing* and *active coping*. A higher level of social identification did not impact well-being or depression through the use of *instrumental* and *emotional support coping*, but through the more frequent use of *positive reframing* and *active coping*. For chronic pain therapy, we propose including modules that foster social identification and a positive stress mindset.

## Introduction

### Background

Chronic pain means persistent or prolonged pain, usually with no or insufficient physical evidence to explain the pain (Dilling & Freyberger, 2019), that has far-reaching consequences for individuals’ private and working lives (e.g., Sagula & Rice, 2004). Chronic pain is one of the most significant causes of suffering worldwide (Lohman et al., 2010). People with chronic pain disease often experience a lack of symptom control, increased risk of unemployment, severe social withdrawal and diagnostic obscurity; all of which can lead to hospitalization (Egloff et al., 2009). While the exact prevalence is unclear, a large proportion of the population suffers from chronic pain, with prevalence estimates varying between 10.1 and 55.2% (Ospina & Harstall, 2002). In the United States, estimates show that 20.4% of adults (i.e. 50.0 million) have chronic pain and 8.0% of adults (19.6 million) have high-impact chronic pain (i.e. interfering with work or life nearly every day; Dahlhamer et al., 2018). In Europe, chronic pain affects approximately 20% of the population (van Hecke et al., 2013).

Since chronic pain is such a prevalent cause of suffering, effective interventions that address its complexity are of great importance. Although pain is described as a biopsychosocial phenomenon, pain research often focuses on the biological aspects of pain, whereas additional efforts are needed to clarify the role of psychological and social factors to improve interventions (Truchon, 2001). Biological, psychological and social aspects not only influence the amount and severity of pain, but pain also produces biological, psychological, and social changes, such as changes in the nervous system pathways (biological), coping efforts and beliefs about pain (psychological), emotional distress of spouses (social) and others that can affect responses to pain (Keefe & France, 1999). One relevant factor for chronic pain patients is their handling of and mindset toward stress. Chronic pain patients subjectively experience stress more frequently compared to people without chronic pain and have an objectively higher stress level, i.e. higher cortisol levels (Van Uum et al., 2008). Strain due to stress, on the other hand, can influence the pain processing of the central nerve system (CNS), which makes a speedy reduction or elimination of the pain unrealistic (Egloff et al., 2009). Chronic pain thus seems to elevate stress levels, which in turn leads to the persistence or even increase of chronic pain. Thereby, research on the so-called stress mindset shows that an individual’s attitude towards stress is important in handling stress and experiencing well-being outcomes (Crum et al., 2013). A stress mindset refers to an individual’s belief about whether stress is perceived to benefit performance, productivity, health and growth (referred to as the stress-is-enhancing mindset) or whether it is more likely to negatively impact these variables (referred to as the stress-is-debilitating mindset; Crum et al., 2013). Initial research into children with chronic pain suggests that chronic pain patients more often have a stress-is-debilitating mindset (Heathcote et al., 2018), which, in turn, may negatively affect their coping behaviors, such as active coping and positive reframing, as previous stress mindset research suggests (Crum et al., 2013, 2017). Positive reframing means trying to look at a situation from a more positive perspective or finding something good in what happened to you, while active coping means focusing on trying and/or taking action to change something about the stressful situation you are in (Knoll et al., 2005). Such coping behavior has been shown to positively affect well-being, leading to, e.g., less substance use or self-harming behavior and more satisfaction with weight, vitality and sleep (Chua et al., 2015).

At the same time, chronic pain patients can cause their spouses, peers and others emotional distress (Keefe & France, 1999), which can result in unresolved social stress for the chronic pain patient. Unresolved social stress, in turn, can be a risk factor for the chronification of the pain (Zimmermann, 2004). Another cause of social stress may be a lack of social identification, which is defined as “the positive emotional valuation of the relationship between self and ingroup” (Postmes et al., 2013, p. 599), where “ingroup” means a social group to which a person belongs. Chronic pain patients frequently withdraw from their social environment (Beeckman et al., 2020; Egloff et al., 2009) and have lower levels of social functioning (Simons et al., 2010) and social competence (Varni et al., 2006), which could lead them to identify less with their social groups. The perceived difference between the impaired self and one’s healthy peers, might also contribute to a lower level of social identification in chronic pain patients. A high level of social identification, on the other hand, is proven to have a beneficial outcome on psychological and physical health (e.g., Postmes et al., 2019; Steffens et al., 2017). Hence, a chronic pain disorder appears to lead to a lower level of social identification, while a high level of social identification, in contrast, would be beneficial for the physical and mental well-being of chronic pain patients. Given that individuals with chronic pain are likely to identify less with their social groups, this might negatively affect their coping behaviors, specifically instrumental and emotional support coping, which, in turn, would negatively affect their well-being. Instrumental support coping concerns either trying to ask or actually asking others for help or advice, while emotional support coping includes receiving encouraging support or sympathy from others or being comforted by others (Knoll et al., 2005). If chronic pain patients do not socially identify with their peers, they are probably less likely to seek instrumental or emotional support from them. However, using instrumental or emotional support coping would benefit their well-being (e.g., Crabtree et al., 2010; Sani et al., 2012).

This study aims to advance the biopsychosocial model of chronic pain by examining chronic pain patients’ stress mindset and social identification as important mechanisms in explaining why those participants engage in less beneficial coping behavior, which, in turn, influences their well-being. In doing so, we develop a model in which participants’ stress mindsets and social identification as well as their coping behavior mediates the relationship between chronic pain and well-being. In this study, we focus on general well-being and depressive symptoms as indicators of health, because we want to examine one positive and one negative indicator for people’s well-being, which is defined as “optimal psychological functioning and experience” (Ryan & Deci, 2001, p. 142), while depression means that an individual suffers from a range of depressive symptoms, e.g., depressed mood, loss of interest and enjoyment, reduced energy and diminished activity (Dilling & Freyberger, 2019). By examining and highlighting the pivotal role of chronic pain patients’ stress mindset and social identification, this study has important practical implications: To interrupt the upholding mechanism of chronic pain and to improve the well-being of chronic pain patients. Therapeutic interventions comprising stress education and how to foster social identification may be effective.

### Theory and Hypothesis Development

#### Chronic Pain and Well-Being

Chronic pain often leads to insomnia, substance abuse, trust issues and problems in interactions with family and/or friends, as well as to losses in relationships, at work or in other areas of life (Sagula & Rice, 2004). It can also impede mobility and lead to a loss of physical strength, an impaired immune system, bad appetite, poor diet, dependence on medicine and/or caretakers/family, excessive use of the healthcare system, poor job performance or inability to work, isolation from society or family, anxiety, acrimony, frustration, depression or even to suicide (Niv & Devor, 2001). Altogether, chronic pain can create a negative downward spiral (Sagula & Rice, 2004) that not only affects physiological, but also psychological and social aspects (e.g., Lohman et al., 2010). Hence, it is not surprising that Gureje et al. (1998) find that chronic pain is associated with psychological illnesses, such as depression or anxiety disorders.

Chronic pain should therefore also affect a person's overall well-being, i.e., their “optimal psychological functioning and experience” (Ryan & Deci, 2001, p. 142). This definition is in line with that of the World Health Organization (WHO; Huber et al., 2011), which emphasizes that all three aspects of well-being—physical, psychological and social—are relevant. Indeed, Björnsdóttir et al. (2014) show that chronic pain patients report worse quality of life and less well-being compared to people without chronic pain. In summary, the literature implies that chronic pain patients have lower general well-being and poorer mental health than people without chronic pain. As depression is one of the most frequent mental illnesses (Lehtinen & Joukamaa, 1994) and therefore a common cause of bad mental health, we used depression as a negative indicator for well-being. Therefore, we predict:

##### Hypothesis 1:

Chronic pain patients report lower general well-being and more depression than people without chronic pain.

#### Stress Mindset and Chronic Pain

Chronic pain patients subjectively experience more stress than people without chronic pain, and hair sample analyses reveal that they have higher cortisol levels (Van Uum et al., 2008). Flor et al. (1985) show that patients with chronic back pain have higher muscular reactivity in their backs and return to the reactivity baseline slower when they discuss personally stressful situations. This was not the case for patients with chronic pain in other body parts. Those findings imply that chronic pain patients’ stress-related responses might play an important role for handling their chronic pain and that those patients show significant differences concerning stress compared to people without chronic pain. Stress is defined as the “relationship between the person and the environment that is appraised by the person as taxing or exceeding his or her resources and endangering his or her well-being” (Lazarus & Folkman, 1984, p. 21). Therefore, stress is “the experience of encountering or anticipating adversity in one’s goal-oriented efforts” (Carver & Connor-Smith, 2010, p. 684).

Despite the adaptive nature of the human body’s physiological response to stress (Sapolsky, 1996), the media and popular literature largely portray stress as something negative (Crum et al., 2013). However, individuals’ attitudes towards stress, i.e., their stress mindset, significantly influence their stress response. According to Crum et al. (2013), individuals differ whether they have a stress-is-enhancing mindset (SIE) or a stress-is-debilitating mindset (SID). An SIE mindset describes the perception that stress leads to better performance, productivity, health, well-being, learning and growth. An SID mindset, on the other hand, comprises the perception that stress negatively influences those variables (Crum et al., 2013). Individual’s mindsets affect their evaluation (e.g., Taylor & Gollwitzer, 1995), their behavior (e.g., Liberman et al., 2004) and their health (e.g., Crum & Langer, 2007). An SIE mindset influences evaluation, as it leads to a higher attention bias for positive stimuli and a higher level of cognitive flexibility (Crum et al., 2017), which might influence coping behaviors like positive reframing that could result in a better health.

Even though previous research shows that people with and without chronic pain differ in the stress they experience, it is unclear if there are also differences in their stress mindsets. Ben-Avi et al. (2018) assume that it is harder for a person to develop an SIE mindset if they are exposed to chronic stressors. As chronic pain patients are exposed to chronic stressors from their chronic pain and because they have heightened stress levels compared to those without chronic pain (Van Uum et al., 2008), the elevated strain due to their heightened stress likely leads to a negative view of stress, i.e. an SID mindset. Indeed, initial evidence supports this proposition: Heathcote et al. (2018) show that children with chronic pain are more likely to have an SID mindset than an SIE mindset compared to children without chronic pain. Within those children with chronic pain, those who had an SID mindset showed significantly more pain-related distress, such as fear of pain and pain catastrophizing and more functional constraints, such as activity limitations. Building upon this previous research, we predict:

##### Hypothesis 2:

Chronic pain patients report a lower SIE mindset compared to people without chronic pain.

#### Relationship Between Stress Mindset, Coping and Well-Being

Crum et al. (2013) show that the stress mindset is a significant predictor for health and life satisfaction. They argue that individuals with an SIE mindset report better health than those with an SID mindset. This could be especially relevant for chronic pain patients. It has been shown that having an SIE mindset leads to more positive affect (Crum et al., 2017). Positive affect on the other hand, was identified as a predictor for a lower pain level in chronic pain patients, while negative affect leads to a higher pain level (Zautra et al., 2005). Therefore, an SIE mindset may positively impact the pain level and well-being of chronic pain patients.

The question is which mechanism beyond affect is relevant for the influence of the stress mindset on health. Crum et al. (2013) suggest different associations of the stress mindset with motivational and physiological processes as a theoretical basis of this effect. They propose that an individual’s stress mindset influences how that person views stress psychologically as well as how they react to stress behaviorally. How a person responds to stress is described as coping, which is defined as “constantly changing cognitive and behavioral efforts to manage specific external and/or internal demands that are appraised as taxing or exceeding the resources of the person” (Lazarus & Folkman, 1984, p. 141). Thus, coping is necessary for handling stressful situations (Folkman et al., 1986; Knoll et al., 2005) and it can improve well-being (e.g., Chua et al., 2015). Coping behaviors can be maladaptive (e.g., denial or avoidance) or adaptive (e.g., active coping or positive reframing) (Folkman & Lazarus, 1985). Crum et al. (2013) argue that a person with an SIE mindset would have the primary motivation to accept stress and utilize its positive consequences. Thus, this person would rather use a behavior that satisfies the requirements, the value or the goal, which underlie the stressful situation. Handling of stress in this way can best be classified as active coping, which means focusing on trying and/or taking action to change something about the stressful situation you are in (Knoll et al., 2005).

The literature implies that the stress mindset may also affect the coping behavior positive reframing, as the stress mindset influences cognition and affect. An SIE mindset leads to more positive affect, more attention to positive stimuli and higher level of cognitive flexibility (Crum et al., 2017). This could lead to people with SIE mindsets being more likely to positively reframe, as this coping behavior is based on such described cognitive processes. Specifically, positive reframing comprises looking at a situation from a more positive perspective or finding something good in what happened to you (Knoll et al., 2005). An SID mindset, however, may worsen those cognitive and affective outcomes (Crum et al., 2017). Hence, we predict:

##### Hypothesis 3:

An SIE mindset is associated with a more frequent use of positive reframing and active coping.

The beneficial impact of coping behaviors on well-being is well documented (e.g., Chua et al., 2015). Positive reframing is also a key psychotherapeutic technique in treating several mental illnesses, especially depression (e.g., Conoley & Garber, 1985). Therefore, we predict:

##### Hypothesis 4:

A more frequent use of positive reframing and active coping is related to a higher general well-being and less depression.

Combining these two hypotheses, we expect that chronic pain patients—compared to people without pain—report a lower SIE mindset, leading to less use of positive reframing and active coping, which, in turn, relates to their well-being. Thus, we predict:

##### Hypothesis 5:

The relationship of chronic pain with general well-being and depression is mediated by (1) an individual’s stress mindset and (2) their use of positive reframing and active coping.

#### Social Identification

Chronic pain is not only influenced by biological and psychological factors, but also by social factors (Ehde et al., 2014; Turk & Okifuji, 2002). One crucial social driver for one’s well-being is social identification (e.g., Steffens et al., 2017), which refers to a person’s relationship to a social group and the positive emotional evaluation of this relationship with a person or a group (Postmes et al., 2013). Meta-analyses find that greater levels of social identification are associated with less depression (Postmes et al., 2019) as well as with better psychological and physical health (Steffens et al., 2017). Social identification could also be an important factor for chronic pain patients. The same area of the brain that is responsible for the degree of pain a person feels—the cingulate gyrus (ACC)—is also responsible for the feeling of loneliness (Spitzer & Bonenberger, 2012). This means that when a person feels pain, the same brain area is activated as when they feel lonely or socially isolated. Researchers also assume that chronic pain can lead to loneliness, which can ultimately lead to depression (Spitzer & Bonenberger, 2012). Indeed, research shows that chronic pain patients have lower levels of social functioning (Simons et al., 2010), fewer social skills (Varni et al., 2006), and that they withdraw frequently from social interactions (Beeckman et al., 2020; Egloff et al., 2009). Haslam et al. (2016b) argue that difficult life transitions, like being diagnosed with a disease, can cause the loss of social relationships, which appears to apply for chronic pain patients. If chronic pain patients, on the one hand, withdraw from their social relationships and belong to fewer groups, they can also identify with fewer such groups, which will result in them having a lower level of social identification compared to people without chronic pain. On the other hand, chronic pain patients might view their belonging to social groups as less favorable, due to their chronic pain, which may also decrease their social identification. Klapow et al. (1995) show that chronic pain patients are less satisfied with the social support they receive from their environment compared to pain patients with a lower pain level. Such dissatisfaction may lower their social identification, because it might lead to a more negative emotional evaluation of the relationship. Furthermore, the negative aspects of a social relationship cloud the positive qualities in difficult times (Davis et al., 2004). It may be possible, that the greater strain experienced by chronic pain patients (e.g., Lohman et al., 2010) causes negative aspects of social relationships to be more ostensible in their perception. This would in turn also lead to a more negative emotional evaluation of those social relationships and thus to a lower level of social identification. Thus, we predict:

##### Hypothesis 6:

Chronic pain patients report a lower level of social identification compared to people without chronic pain.

#### Relations Between Social Identification, Coping and Well-Being

Social identification is an important basis for social support, which buffers stress (Haslam et al., 2005), protects mental health (Sani et al., 2012), and increases well-being (Crabtree et al., 2010). This could be especially relevant for chronic pain patients, who—as Hypothesis 1 argues—appear to already have an impaired well-being (e.g., Björnsdóttir et al., 2014) and have a lower level of social identification—as the previous section argues.

Following arguments of Haslam et al. (2016c), if individuals identify with their social groups, they are likely to receive more social support from them (Haslam et al., 2005). Seeking social support can be a coping behavior (Knoll et al., 2005). Social support can be divided into instrumental and emotional support—the former concerns either trying to ask or actually asking others for help or advice, while the latter comprises receiving encouraging support or sympathy from others or to be comforted by others (Knoll et al., 2005). If people identify less with social groups, they will likely seek and receive less instrumental and emotional support (Haslam et al., 2005), and use these coping behaviors less often. For example, if a person with chronic pain does not identify with another person or a group, they are probably less likely to ask to be taken to the doctor (instrumental support) or to ask them to come over to talk on days when the pain is especially strong (emotional support). Thus, they would be less likely to receive support. Therefore, we predict:

##### Hypothesis 7:

A higher level of social identification is associated with a more frequent use of instrumental and emotional support coping.

Social support positively influences mental health (Sani et al., 2012) and well-being (Crabtree et al., 2010). Research shows that social support is greatly relevant for well-being, especially in chronic pain patients. For example, active or passive support of another person can reduce pain (Brown et al., 2003). Likewise, Master et al. (2009) provide an overview of studies, which document the pain-attenuating effect of social relationships and show that even the mental representation of a loved, supporting person (e.g., looking at a picture of this person) can reduce pain. The importance of social identification and social support for pain reduction has also been shown experimentally. Platow et al. (2007) show that people who experience experimental pain in a laboratory setting (immersing a hand into a bath of ice water) report less pain when they receive support from a group member with whom they identify, than when they receive support from a group with which they do not identify. Hence, we predict:

##### Hypothesis 8:

A more frequent use of instrumental and emotional support coping is related to a higher general well-being and less depression.

In summary, we propose that chronic pain patients (compared to people without pain) report a lower level of social identification, which influences the amount of instrumental and emotional support that they seek and receive, and, ultimately relates to their well-being. Hence, we predict:

##### Hypothesis 9:

The relationship of chronic pain with general well-being and depression is mediated by (1) an individual’s social identification and (2) their use of instrumental and emotional support coping.

**Fig. 1:**
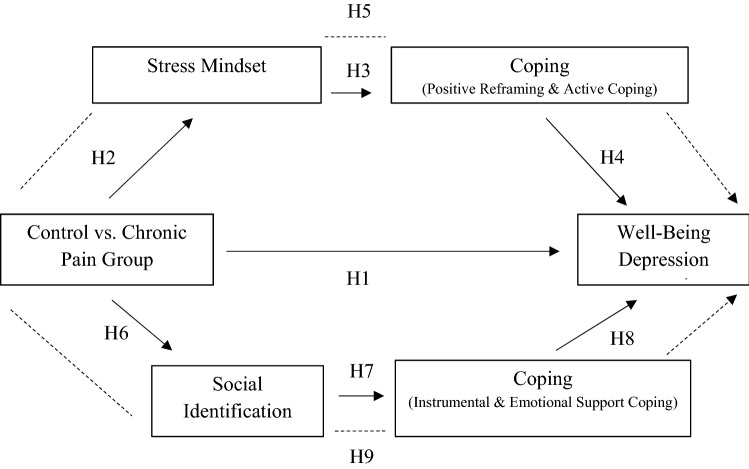
Theoretical model of hypotheses. H5 refers to the indirect relationship of the chronic pain on well-being through the stress mindset and coping (positive reframing and active coping) in terms of a sequential mediation. H9 refers to the indirect relationship of the chronic pain on well-being through social identification and coping (instrumental and emotional support coping) in terms of a sequential mediation

As the hypotheses contain a comparison between chronic pain patients and people without chronic pain, a quasi-experimental study design was implemented. Figure 1 shows an overview of the hypotheses.

## Methods

### Participants and Procedure

The survey was part of a study project and we received ethical approval by our institutional ethics committee to conduct this study. Power analysis conducted in G Power (MacUpdate, 2020) and R (Version 4.1.2, R Core Team, 2021) with packages pwr (Champely et al., 2020) and lavaan (Rosseel, 2012) revealed sample sizes of as low as n = 38^1^ to as high as n = 700.^2^ We recruited more than that, in case we needed to exclude some participants (due to missing values, etc.). Post-hoc power and sensitivity analyses revealed standardized single indirect, doubly mediated effects of as low as 0.005 to be detectable with the final sample size in a manifest mediation model and as low as 0.008 in a latent mediation analysis at a power of 0.90. A quasi-experimental design was used, as chronic pain patients and people without chronic pain (control group) are already existing and naturally occurring groups. Participants received no payments etc. for participating, but psychology students that participated in the control group could receive university credit points. Participants were recruited in the time of February till April 2020. We recruited the chronic pain group through professional institutions that treat chronic pain, as well as through social networks specifically for chronic pain patients (e.g. online self-support groups). The control group was recruited via social networks only. A total of 1319 people answered the online questionnaire. Before answering the questionnaire, participants were informed about the study online via the questionnaire link and they could only proceed to the questionnaire, if they indicated that they had read the study information, agreed to the terms and conditions and gave their consent to participate in this study. Data was collected anonymously, meaning there can be no inference made from the provided data to the participants.

In the chronic pain group, participants had to indicate whether they had a chronic pain disorder and could specify which one (e.g. Fibromyalgia, migraine, etc.) in an open question. The participants in both study groups had to be at least 18 years old. Participants in the control group, who indicated having chronic pain were excluded. They could not be transferred to the chronic pain group, as they answered a version of the questionnaire that did not include the pain-related questions. We removed 32 participants because of this criterion. We also excluded those participants that finished the questionnaire in less than 50% of the average completion time, as we assumed that they answered the questionnaire too hastily in such little time. For this criterion, we chose the median instead of the mean, since respondents had the option to pause their participation and continue it later. This sometimes resulted in long processing times, which would have biased the mean. The median duration was 14.58 min. Therefore, we excluded 27 respondents who finished the questionnaire in less than 7.5 min. Another 20 participants were excluded for producing invalid data or failing to meet the participation criteria (e.g., were under 18 years old). The final sample size was *N* = 1240 with *N* = 465 participants in the chronic pain group and *N* = 775 participants in the control group. The control group consisted of 88.26% females, 11.35% males and 0.39% non-binary people, with a mean age of *M* = 30.67 (SD 9.10, ranging from 18 to 76 years). In the control group, the three most common highest educational achievements were a bachelor degree (40.1%), a master degree (22.1%) and a High School leaving certificate (21.3%). The chronic pain group consisted of 95.05% women, 4.52% men and 0.43% non-binary people, with a mean age of *M* = 45.77 (SD 12.40, ranging from 20 to 80). In the chronic pain group, the three most common highest educational achievements were a completed apprenticeship (44.7%), a General Certificate of Secondary Education (13.8%) and a master degree (10.5%). Regarding ethnicity 92.7% of all participants were German, the other 7.3% had different nationalities, like Austrian, Turkish, Bosnian, etc. In the overall sample, the three most common highest educational achievements were a bachelor degree (27.4%), a completed apprenticeship (23.6%) and a master degree (17.7%). The most common chronic pain condition in our sample was Fibromyalgia, the second most common was chronic pain disorder/somatoform disorder/complex regional pain syndrome and the third most common was endometriosis/period pain.^3^ Participants in the chronic pain group indicated that they had experienced the chronic pain for 12 years on average (*M* = 11.78 years, SD 10.24 years) and that they feel the pain either several times a week or daily (*M* = 5.65, SD 0.82 on a scale from 1 to 6). The average intensity of the pain (*M* = 7.48, SD 1.52 on a scale from 1 to 11), the social constraints due to the pain (*M* = 8.02, SD 2.06 on a scale from 1 to 11), as well as the general strain because of the pain (*M* = 8.48, SD 1.83 on a scale from 1 to 11) were all rated as rather high.

### Measures

#### Stress Mindset

We measured this construct using the eight-item Stress Mindset Measure (SMM) developed by Crum et al. (2013). On a 5-point scale from 1 = *Strongly disagree* to 5 = *Strongly agree*, participants rated items like “The effects of stress are positive and should be utilized” or “The effects of stress are negative and should be avoided”. The scale showed a reliability of $\omega$ = 0.91 in our sample. Higher values on this scale indicate an SIE mindset. Crum et al. (2013) demonstrate the discriminant and criterion validity of this measure.

#### Social Identification

To measure how strong participants identify with their social groups, we used a shortened form of the Social Identity Map approach from Haslam et al. (2016a) with eight items. Participants were told that every group can count as a social group (e.g., based on nationality, a hobby, a sport, an occupation, etc.), using the definition of Haslam et al. (2016a). Afterwards, participants were asked to name their social groups (up to eight groups) and to rate each one on a scale from 1 to 10 regarding how positive being a part of that social group makes them feel. Lower values indicate a lower level of positive feeling about the group membership and hence a lower level of social identification. Social identification was calculated by computing a sum score for all groups, with a minimum of 0 and a maximum of 80. The scale showed a reliability of $\omega$ = 0.84 in our sample. Participants who stated that they did not have any social contacts were manually assigned the value 0 for every social variable. Participants who did not provide any value on the social variables above were also manually assigned the value 0.

#### Coping

This construct was assessed using the 28-item German version of the Brief COPE based on Knoll et al. (2005), which Carver (1997) originally published in English. We used the following scales of the Brief COPE: *Positive Reframing* ($\omega$ = 0.75),^4^*Active Coping* ($\omega$ = 0.76), *Instrumental Support* ($\omega$ = 0.84) and *Emotional Support* ($\omega$= 0.75). The items are rated on a 4-point scale from 1 = *I haven’t been doing this at all* to 4 = *I’ve been doing this a lot*. Sample items are “I take action to try to make the situation better” (Active Coping), “I try to see it in a different light, to make it seem more positive” (Positive Reframing), “I try to get advice or help from other people about what to do” (Instrumental Support) or “I get comfort and understanding from someone” (Emotional Support). Amoyal et al. (2016) show that the Brief COPE is a valid and reliable instrument even in clinical samples (i.e. liver transplant patients) and they demonstrate construct validity within this sample.

#### Subjective Psychological Well-Being

To measure participants’ *general well-being*, we used the five-item Well-Being Index (WHO-5) based on Brähler et al. (2007). The participants rated items such as “Over the past 2 weeks I have felt cheerful and in good spirits” and “Over the past 2 weeks I have felt calm and relaxed” on a 6-point scale from 1 = *At no time* to 6 = *All of the time*. The scale showed a reliability of $\omega$ = 0.89 in our sample. According to Brähler et al. (2007), the WHO-5 shows satisfactory psychometric qualities regarding reliability and construct validity. We also used the nine-item Patient Health Questionnaire (PHQ-9) based on Kroenke and Spitzer (2002) to measure *depression* as a negative indicator of psychological well-being. Sample items are “Over the last 2 weeks, I have been bothered by little interest or pleasure in doing things” or “Over the last 2 weeks, I have been bothered by feeling down, depressed, or hopeless”, which were rated on a 4-point scale from 1 = *Not at all* to 4 = *Nearly every day*. In our sample, the scale showed a reliability of $\omega$ = 0.86. The PHQ-9 is a reliable and valid measure of depression severity (Kroenke et al., 2001).

We assume that there is a given comparability of assessment methods across the two study groups, as all of those concepts concern all human beings to some extent and, for example the Stress Mindset Measure and the Brief COPE have already been validated in chronic pain or other clinical samples (Amoyal et al., 2016; Heathcote et al., 2018).

## Data Analysis

Latent mediation analyses were conducted in Mplus (Muthén & Muthén, 1998–2011), using all items of a scale as indicators for their respective latent variables. This resulted in a model with 38 manifest and 8 latent variables. The dichotomous variable indicating chronic pain group membership was included as the 39^th^ manifest variable for the mediation model. The mediation model was simultaneously analyzed for all mediators (stress mindset, social identification, and the four coping behaviors) and outcomes (general well-being and depression). The mediators as well as the outcome variables were allowed to covary. Using modification indices we identified residual correlations between items 2, 6, and 9 as well as items 3 and 4 to counteract the poor fit of the unidimensional PHQ measurement model. From the content of the items, those correlations appear sensible, as item 2, 6 and 9 concern negative affect whereas item 3 and 4 concern feeling tired. Boxplot analyses revealed four outliers. Analyses were calculated with and without those outliers and showed no difference in the results. Therefore, outliers were included in the analyses. Full information maximum likelihood as implemented in Mplus was used to handle missing data. There was not much data missing and where it was missing, it was mostly not at random, as the most missing data occurred in the variables asking about social groups. If a participant for example only had two social groups and left the space for the remaining six social groups open, all of those remaining variables concerning social groups were coded as missing data.

Bootstrapping was performed with 10,000 samples to acquire trustworthy confidence intervals for all coefficients of the latent mediation model. Due to the asymmetric distribution of indirect effects, 95% bootstrapped confidence intervals are used as the basis for inference instead of *p*-values.

## Results

### Descriptive Statistics

Table 1 presents the means, standard deviations and correlations of the manifest scale variables, as well as the scale reliabilities, which range between 0.75 and 0.91, indicating acceptable or good consistency.

**Table 1:** Means (M), standard deviations (SD), correlations and reliabilities of the variables

Variables	M	SD	1	2	3	4	5	6	7	8	9	10	11	12	13	14	15
1. Age	36.33	12.76	–														
2. Gender	–	–	− 0.05	–													
3. Control vs. pain group	–	–	0.57**	− 0.11**	–												
4. Stress mindset	2.42	0.81	− 0.26**	0.05	− 0.42**	(0.91)											
5. Social identification	29.66	15.70	− 0.20**	− 0.02	− 0.30**	0.23**	(0.84)										
6. Coping: positive reframing	5.06	1.64	− 0.13**	0.06*	− 0.22**	0.22**	0.18**	(0.75)									
7. Coping: active coping	5.77	1.48	0.05	− 0.02	0.02	0.09**	0.11**	0.38**	(0.76)								
8. Coping: instrumental support	5.43	1.71	− 0.17**	− 0.02	− 0.13**	0.08**	0.17**	0.27**	0.36**	(0.84)							
9. Coping: emotional support	5.68	1.62	− 0.27**	− 0.03	− 0.28**	0.18**	0.28**	0.30**	0.26**	0.57**	(0.75)						
10. Well-being (WHO)	14.82	5.45	− 0.33**	0.07*	− 0.57**	0.43**	0.34**	0.41**	0.23**	0.21**	0.32**	(0.89)					
11. Depression (PHQ) (as negative indicator for well-being)	19.28	6.06	0.34**	− 0.10*	0.62**	− 0.43**	− 0.29**	− 0.34**	− 0.16**	− 0.15**	− 0.29**	− 0.79**	(0.86)				
12. Endurance of pain	141.37	122.91	0.34**	0.00	–	− 0.05	− 0.01	0.04	0.05	− 0.09	− 0.10*	0.01	0.04	–			
13. Frequency of pain	5.65	0.82	0.24**	0.05	–	− 0.11*	0.01	0.01	0.07	− 0.04	− 0.06	− 0.19**	0.26**	0.14**	–		
14. Average pain intensity	7.48	1.52	0.00	− 0.12**	–	− 0.22**	− 0.06	− 0.12**	− 0.01	0.01	− 0.01	− 0.26**	0.28**	0.04	0.03	–	
15. Strain due to pain	8.48	1.83	− 0.05	− 0.05	–	− 0.29**	− 0.17**	− 0.18**	0.01	0.05	− 0.08	− 0.44**	0.44**	0.01	0.09	0.35**	–

*N* = 1240; for the variables 13–15: *N* = 465 (only chronic pain patients); for variable 12: *N* = 436 (due to missing values). Omega is indicated in the brackets

*p < .05; **p < .01 (two-tailed). Higher values indicate a higher peculiarity; for the stress mindset higher values indicate a stress-is-enhancing mindset, lower values indicate a stress-is-debilitating mindset; Control group = 0, Pain group = 1; Gender: 1 = female, 2 = male, 3 = diverse. Variable 4 is a mean score; variables 5–11 are sum scores; variable 12 is indicated in months

### Hypothesis Testing

The full measurement model including all variables and free covariance structures between latent variables fit the data adequately ($\chi$^2^_(633)_ = 2082.120; *p* < .001; CFI = 0.933, TLI = 0.923, RMSEA [90% C.I. 0.041–0.045] = 0.07, SRMR = 0.048). Misfit was indicated mainly by the two comparative fit indices, hinting at relatively low correlations between the manifest variables included in the model.

We estimated the path coefficients of the predicted paths (see Table 2). Standardized coefficients and their confidence intervals are mentioned in the tables only. Due to the boostrapping performed, 95% confidence intervals are reported instead of *p*-values. The analyses support Hypothesis 1, predicting that chronic pain patients report less general well-being (Total effect: *b* = − 1.365, 95% CI [− 1.471; 1.262], direct effect: *b* = − 0.969, 95% CI [− 1.102; − 0.836]) and more depression (Total effect; 1.001, 95% CI [0.920; 1.083], direct effect: *b* = 0.773, 95% CI [0.680; 0.868]) than people without chronic pain. In addition, chronic pain patients (compared to people without chronic pain), score lower on the stress mindset scale, indicating less of a stress-is-enhancing mindset (*b* = − 0.788, 95% CI [− 0.890; − 0.686]), supporting Hypothesis 2. As Hypothesis 3 predicts, a stress-is-enhancing mindset was associated with a more frequent use of the coping behaviors positive reframing (*b* = 0.145, 95% CI [0.080; 0.205]) and active coping (*b* = 0.099, 95% CI [0.051; 0.147]). A more frequent use of positive reframing and active coping was related to higher general well-being (for positive reframing: *b* = 0.389, 95% CI [0.277; 0.510]; for active coping: *b* = 0.207, 95% CI [0.134; 0.278]) and less depression (for positive reframing: *b* = − 0.164, 95% CI [− 0.237; − 0.094]; for active coping: *b* = − 0.117, 95% CI [− 0.207; − 0.026]), providing evidence for Hypothesis 4. The analyses of the indirect effects revealed that the relationship between chronic pain and well-being is mediated by (1) individual’s stress mindset and (2) positive reframing and active coping (indirect effect on general well-being via positive reframing and stress mindset: *b* = − 0.045, 95% CI [− 0.073; − 0.024], indirect effect on general well-being via active coping and stress mindset: *b* = − 0.016, 95% CI [− 0.034; − 0.005], indirect effect on depression via positive reframing and stress mindset: *b* = 0.019, 95% CI [0.009; 0.033], indirect effect on depression via active coping and stress-mindset: *b* = 0.010, 95% CI [0.009; 0.033]; see Table 3). Thus, Hypothesis 5 is supported. Chronic pain patients report a lower level of social identification compared to people without chronic pain (*b* = − 0.883, 95% CI [− 1.125; − 0.686]), in line with Hypothesis 6. Results also support Hypothesis 7, which predicts that a higher level of social identification is related to a more frequent exertion of the coping behaviors instrumental (*b* = 0.042, 95% CI [0.004; 0.086]) and emotional support coping (*b* = 0.163, 95% CI [0.119; 0.217]). However, we could find no support for Hypothesis 8, which posits that the more frequent use of instrumental and emotional support coping is associated with a greater well-being: Neither instrumental nor emotional support coping showed a relationship with general well-being (for instrumental support coping: *b* = − 0.015, 95% CI [− 0.162; 0.128] and for emotional support coping: *b* = 0.034, 95% CI [− 0.165; 0.234]). Instrumental support coping shows no significant relationship for depression as an outcome variable (*b* = 0.066, 95% CI [− 0.029; 0.165]), as does emotional support coping (*b* = − 0.034; 95% CI [− 0.165; 0.234]). Hence, Hypothesis 9 is also not supported: Social identification and emotional support coping do not significantly mediate the relationship between chronic pain and depression (indirect effect: *b* = − 0.005, 95% CI [− 0.035; 0.025). None of the remaining indirect relationships from chronic pain to both well-being outcomes via (1) social identification and (2) instrumental and emotional support coping were significant (indirect effect on general well-being via instrumental support coping and social identification: *b* = 0.001, 95% CI [− 0.005; 0.008], indirect effect on general well-being via emotional support coping and social identification: *b* = 0.008, 95% CI [− 0.012; 0.029], indirect effect on depression via instrumental support coping and social identification: *b* = − 0.002, 95% CI [− 0.010; 0.000]).

**Table 2:** Mediation model

	Stress mindset *b* (CI); *β*	Social identification *b* (CI); *β*
Control vs. pain group	− **0.79** (− 0.89; − 0.67); − 0.44	− **0.88** (− 1.12; − 0.69); − 0.30
*R*^2^	0.20	0.09

	Positive reframing *b* (CI);* β*	Active coping *b* (CI); * β*
Control vs. pain group	− **0.23** (− 0.34; − 0.13); − 0.16	**0.11** (0.02; 0.19); 0.09
Stress mindset	**0.15** (0.08; 0.20) 0.18	**0.10** (0.05; 0.15); 0.15
*R*^2^	0.08	0.02

	Instrumental support *b* (CI); * β*	Emotional support *b* (CI); * β*
Control vs. pain group	− **0.18** (− 0.28; − 0.08); − 0.11	− **0.32** (− 0.42; − 0.23); − 0.24
Social identification	**0.04** (0.00; 0.09); 0.08	**0.16** (0.12; 0.22); 0.36
R^2^	0.02	0.23

	WHO *b* (CI); * β*	PHQ *b* (CI); *β*
Control vs. pain group	− **0.97** (− 1.10; − 0.84); − 0.44	**0.77** (0.68; 87); 0.53
Stress mindset	**0.21** (0.13; 0.28); 0.17	− **0.14** (− 0.19; − 0.10) − 0.17
Social identification	**0.10** (0.04; 0.18); 0.14	− **0.06** (− 0.11; − 0.02); − 0.12
Positive reframing	**0.39** (0.28; 0.51); 0.26	− **0.16** (− 0.24; − 0.09); − 0.17
Active coping	**0.21** (0.06; 0.35); 0.11	− **0.12** (− 0.21; − 0.03); − 0.09
Instrumental support	− 0.02 (− 0.16; 0.13); − 0.01	0.07 (− 0.03; 0.16); 0.07
Emotional support	0.03 (− 0.16; 0.23); 0.02	− 0.05 (− 0.19; 0.08); − 0.05
*R*^2^	0.56	0.58

Unstandardized coefficients reported (b). We use standardized *β* as a measure for effect size. Effects are bold if the 95% confidence interval does not include 0, indicating significant effects at *p* = .05

*WHO* general well-being, *PHQ* depression; *Control group* 0, *Pain group* 1, *SE* standard error, *CI* confidence interval (lower 2.5%, upper 2.5%)

**Table 3:** Indirect effects for the mediation model

Indirect effects			
	Estimate	CI	*β*
Total indirect effect			
For WHO	− **0.397**	[− 0.501; − 0.299]	− 0.180
For PHQ	**0.229**	[0.163; 0.296]	0.157
Indirect effect			
Gr → SM → PR → WHO	− **0.045**	[− 0.073; − 0.024]	− 0.020
Gr → SM → AC → WHO	− **0.016**	[− 0.034; − 0.005]	− 0.007
Gr → SM → PR → PHQ	**0.019**	[0.009; 0.033]	0.013
Gr → SM → AC → PHQ	**0.009**	[0.002; 0.020]	0.006
Gr → Soc → IS → WHO	0.001	[− 0.005; 0.008]	0.000
Gr → Soc → ES → WHO	− 0.005	[− 0.035; 0.025]	− 0.002
Gr → Soc → IS → PHQ	− 0.002	[− 0.010; 0.000]	− 0.002
Gr → Soc → ES → PHQ	0.008	[− 0.012; 0.029]	0.005
Total effect			
Gr → WHO	− **1.365**	[− 1.471; − 1.262]	− 0.620
Gr → PHQ	**1.001**	[0.920; 1.083]	0.688

Unstandardized coefficients reported. We use standardized *β* as a measure for effect size. Effects are bold if the 95% confidence interval does not include 0, indicating significant effects at *p* = .05

*Gr* control (= 0) vs. pain group (= 1), *SM* stress mindset, *Soc* social identification, *PR* positive reframing, *AC* active coping, *IS* instrumental support, *ES* emotional support, *WHO* general well-being, *PHQ* depression, *SE* standard error, *CI* confidence interval (lower 2.5%, upper 2.5%)

We conducted exploratory analyses to test a modified model including the link of social identification with positive reframing and active coping, in addition to the paths described above. We tested this model because previous studies on women with impairments, such as reduced activity and depression due to premenstrual syndrome (PMS), suggest that an increase in social support through self-help groups and thus greater levels of social identification with women with the same condition can positively affect positive reframing as well as activation (and thus maybe also more active coping) in these women, which also resulted in less impairment and depression (Morse, 1997). Model comparisons revealed the constraints imposed on the mediation paths in the previous model to be too restrictive (Δ*χ*^2^_(4)_ = 32.75, *p* < .001; AIC = 24.75, BIC = 4.26). The modified model shows similarly adequate fit indices ($\chi$^2^_(663)_ = 2333.30, *p* < .001; CFI = 0.923, TLI = 0.917, RSMEA = 0.045 [90% CI 0.043; 0.047], SRMR = 0.048). We find that higher level of social identification is associated with more positive reframing (*b* = 0.091, 95% CI [0.048; 0.142]) and with more active coping (*b* = 0.076, 95% CI [0.042; 0.116]). Both of those coping behaviors are positively related to well-being (positive reframing on general well-being: *b* = 0.378, 95% CI [0.269; 0.501], active coping on general well-being: *b* = 0.199, 95% CI [0.056; 0.338]) and negatively related to depression (positive reframing on depression: *b* = − 0.159, 95% CI [− 0.231; − 0.091], active coping on depression: *b* = − 0.111, 95% CI [− 0.203; − 0.021]). The indirect relationships between chronic pain and both well-being variables via (1) social identification and (2) positive reframing as well as active coping were significant (indirect effect on general well-being via positive reframing and social identification: *b* = − 0.031, 95% CI [− 0.053; − 0.016], indirect effect on general well-being via active coping and social identification: *b* = − 0.013, 95% CI [− 0.028; − 0.004], indirect effect on depression via positive reframing and social identification: *b* = 0.013, 95% CI [0.006; 0.024], indirect effect on depression via active coping and social identification: *b* = 0.008, 95% CI [0.002; 0.017]), supporting the notion of a mediating effect. Thus, the modified model shows that chronic pain is linked to a lower level of social identification, although a higher level of social identification is related to a more frequent use of the coping behaviors positive reframing and active coping—besides emotional and instrumental support coping—which are both, in turn, related to a better well-being, i.e., better general well-being and less depression (see Table 4 for indirect effect coefficients of the modified model).

**Table 4:** Indirect effects for the modified model

Indirect effects			
	Estimate	CI	*β*
Gr → Soc → PR → WHO	− **0.031**	[− 0.053; − 0.016]	− 0.014
Gr → Soc → AC → WHO	− **0.013**	[− 0.028; − 0.004]	− 0.006
Gr → Soc → PR → PHQ	**0.013**	[0.006; 0.024]	0.009
Gr → Soc → AC → PHQ	**0.008**	[0.002; 0.017]	0.005

Unstandardized coefficients reported. We use standardized *β* as a measure for effect size. Effects are bold if the 95% confidence interval does not include 0, indicating significant effects at *p* = .05

*Gr* control (= 0) vs. pain group (= 1), *SM* stress mindset, *Soc* social identification, *PR* positive reframing, *AC* active coping, *IS* instrumental support, *ES* emotional support, *WHO* well-being index, *PHQ* well-being index (measuring depression), *SE* standard error, *CI* confidence interval (lower 2.5%, upper 2.5%)

## Discussion

This study’s results contribute to the chronic pain literature. They show that chronic pain patients have a worse well-being (H1), a more negative stress mindset (H2) and a lower level of social identification (H6) than people without chronic pain. Having a positive stress mindset is linked to the more frequent use of the adaptive coping behaviors positive reframing and active coping (H3), which, in turn, relate to greater well-being and fewer depressive symptoms (H4). The connection between a chronic pain disorder and well-being as well as depression was sequentially mediated through (1) the stress mindset and (2) coping (positive reframing and active coping) (H5). Having a greater level of social identification is connected with the more frequent use of the adaptive coping behaviors instrumental and emotional support coping (H7). Contrary to our expectations, those two coping behaviors were not related to well-being and depression (H8). Therefore, we found the connection between a chronic pain disorder and well-being not to be sequentially mediated through (1) social identification and (2) instrumental and emotional support coping (H9). Additional analyses reveal however, that a higher level of social identification is linked to the more frequent use of positive reframing and active coping, thus contributing to better well-being and less depression through those coping behaviors.

### Theoretical and Research Implications

Our findings are widely consistent with the literature. We confirm results in a sample of children with chronic pain by Heathcote et al. (2018) for adults with chronic pain, adding upon their results by revealing the negative consequences of a negative stress mindset in chronic pain patients for coping and well-being. We also incorporate social identification research into the field of chronic pain, which is a rarely taken approach, although the former shows its important effects on physical and mental health (e.g., Steffens et al., 2017), which is especially important for people suffering from chronic pain.

The finding that instrumental support coping showed no connection with well-being may be due to our operationalization. To measure instrumental support coping, we used the questions from the Brief COPE questionnaire (Knoll et al., 2005), which asks participants if they had sought help or advice from others. However, we did not ask if they actually *received* helpful advice or help from others, which is supposedly important for a positive influence from instrumental support coping on well-being. Merely asking for help or advice might not lead to better well-being, as this could also cause people to think that they are burdening others or that they are not good or strong or smart enough to deal with their problems on their own. On the other hand, actually receiving instrumental support after asking for it may—in addition to the positive effect of the (material) support itself—increase feelings of self-efficacy, which could contribute to better well-being. Jensen et al. (1991) stress that self-efficacy beliefs are critical for chronic pain patients’ coping efforts. Thus, future studies should include participants’ perceptions of whether they actually received helpful support after seeking it and evaluate whether this influences the relationship between instrumental support coping and well-being. Concerning the finding that emotional support coping showed no connection with well-being, this may also be due to our operationalization. The Brief COPE asks if you received emotional support, however, it does not ask if you were satisfied with the support received. Fernández-Peña et al. (2020) argue that the satisfaction with received support differs according to personal characteristics such as age, pain intensity, the amount of time since the onset of chronic pain, or with characteristics related to the personal network. We did not ask participants if they were satisfied with the support they received, which may influence not only the relationship between emotional but also instrumental support coping and well-being. López-Martínez et al. (2008) find that the satisfaction of chronic pain patients with their received social support is significantly associated with a less depressed mood and lower intensity of pain. Therefore, instrumental and emotional support coping may improve well-being in chronic pain patients, but we simply do not find such a link because we did not account for other important variables, such as self-efficacy beliefs, the actual reception of support or satisfaction with such received support. Future studies may include such factors.

As we recruited chronic pain patients through different channels (virtual self-support groups, in person self-support groups, institutions that treat chronic pain, chronic pain information networks), we assume that our sample is representative for the group of chronic pain patients. In addition, our large sample size might contribute to a higher generalizability and representativeness of our results.

### Practical Implications

Therapeutic methods to treat chronic pain aim at improving coping behaviors and well-being (Egloff et al., 2009). Therefore, our results have important implications for an adjustment of treatment methods, as they show that the stress mindset as well as social identification can play an important role in achieving those two therapeutic goals. Both concepts are—according to our best knowledge—not a part of the treatment process yet. We suggest integrating a module into therapy, which includes education about stress and the stress mindset and that uses cognitive reframing in order to help the patients gain a stress-is-enhancing mindset. Crum et al., 2011) developed a stress mindset training that could serve as a template for developing such a module. This training has already been evaluated and significantly improves health and well-being (Crum et al., 2011).

To improve social identification in chronic pain therapy, we suggest offering the patients participation in a Groups4Health (G4H) training (Haslam et al., 2016a). This training comprises five modules that are held in a group setting. The goal of this training program is to help patients acquire, improve and sustain social group memberships (Haslam et al., 2016a). The G4H training significantly improves mental health, well-being and social connectedness (Haslam et al., 2016b). Participants who complete the G4H training show improvements in depression, anxiety, stress, loneliness, and life satisfaction, which were underpinned by participants’ increased identification with their G4H group as well as with multiple other social groups (Haslam et al., 2016b). Those results are in line with our finding that a greater level of social identification is linked to less depression. Of course, the usage of both suggested treatment programs (stress mindset training and G4H training) in the therapy of chronic pain should be scientifically evaluated regarding its effectiveness.

### Limitations

Although our model explains roughly half of the variation of well-being and depression, there is still substantial remaining variance influenced through variables other than those that we took into account, and which play a role concerning the negative influence of chronic pain on well-being. Future research should pay more attention to potential confounding variables, like for example other or comorbid medical/psychological conditions besides the chronic pain itself, that could influence the outcome variables. In addition, more scientific knowledge about the reasons for individuals to develop an SID mindset would be helpful, in order to deviate potential confounding variables. Traumatic experiences could, for example, be a possible confounding variable in this regard, which we did not pay heed to. Furthermore, probably due to our operationalization, we do not find sufficient evidence that emotional and instrumental support coping have a positive influence on well-being in chronic pain patients, even though the literature suggests this relationship (e.g., López-Martínez et al., 2008). One potential limitation is that we use subjective self-report data, however this is the only reasonable way to measure most of the constructs used. As for measuring the chronic pain condition however, using self-report data is a bigger limitation. We decided for this strategy rather than for a clinical sample (e.g. recruited only in institutions which treat chronic pain conditions), to maximize external validity of our results by gaining a bigger sample size. We did this especially because the purpose of the study was to identify if the hypothesized constructs and effects even do play a role in the target group of chronic pain patients, as integrating those constructs and their relationships with each other into the research field of this target group is a newly taken approach. However, surely our results should be replicated by future studies using a clinical sample (e.g. only recruited in institutions which treat chronic pain conditions) and thus potentially adding more validity to the measure of the chronic pain condition. In fact, using a cross-sectional design restricts the generalizability of our results. The cross-sectional design is especially a limitation in terms of the mediation analysis we conducted, for which timely precedence is a basis assumption. This assumption, however, is rather complicated using cross-sectional data (Cole & Maxwell, 2003). Nevertheless, our study provides an insight into the important role of the stress mindset and social identification for chronic pain patients’ well-being, which can stimulate future research in this scientific field and therefore be a first step towards the improvement of clinical psychological interventions in the medical setting concerning the treatment of chronic pain. Future research should try to replicate our findings using a longitudinal design. In doing so, such future studies should pay attention to recruiting a sample with more different nationalities, as mostly Germans participated in our study, which limits the generalizability of our results for other nationalities. We also had an uneven gender distribution in our sample, as mainly women participated in both of our study groups, which might limit generalizability for men. However, there is a higher prevalence for chronic pain in females (Sjøgren et al., 2009), which might have influenced the uneven gender distribution in our chronic pain group. In our study, the control group was more of an academic group than the chronic pain group. We do not believe that this lowers the generalizability of our results, as it seems to picture the reality. Sjøgren et al. (2009) found a higher prevalence for less years of education in chronic pain patients.

### Conclusion

Chronic pain patients have a worse well-being, a more negative stress mindset and a lower level of social identification than people without chronic pain. A positive stress mindset is linked to better well-being and less depressive symptoms through the more frequent use of positive reframing and active coping. A higher level of social identification is connected to a more frequent use of instrumental and emotional support coping, however, those two coping behaviors do not seem to be related to well-being and depression. Thus, contrary to our expectations, a higher level of social identification did not impact well-being or depression through the use of instrumental and emotional support coping, but through the more frequent use of positive reframing and active coping. More knowledge about potential confounders for those effects is needed and the results should be replicated using a longitudinal study design. Despite these limitations, our study provides an insight into the important role of the stress mindset and social identification for chronic pain patients’ well-being, which can stimulate future research in this scientific field and therefore be a first step towards the improvement of clinical psychological interventions in the medical setting concerning the treatment of chronic pain. In chronic pain therapy, we propose including and evaluating modules that address and improve the stress mindset (e.g., the stress mindset training by Crum et al., 2011) and social identification (e.g., the G4H training by Haslam et al., 2016a).

## Data Availability

The data was not yet uploaded to a public repository. Upon acceptance of the manuscript by the journal, the authors can send the dataset to the reviewers/editors of the journal and/or upload the dataset into a public repository.

## Footnotes

1 Determined as the required sample size to test Hypothesis 1, assuming a large effect akin to Cohen's *d* = 1.

2 Determined via Monte-Carlo simulation as the required sample size to test a specific indirect effect of the group difference in depression mediated via stress mindset and positive reframing postulated in Hypothesis 5.

3 As many participants indicated more than one diagnosis, we decided for such a descriptive, rather than percentage portrayal.

4 The reliabilities indicated in the brackets are drawn from our current sample.

## Code Availability

Not applicable (see above).
